# Use of Autologous Exosomes to Enhance Oocyte Maturation in Patients with Diminished Ovarian Reserve: A Prospective, Randomized Clinical Trial (Pilot Study)

**DOI:** 10.3390/bioengineering13080875

**Published:** 2026-07-29

**Authors:** Carmen Teresa Navarro Arrieta, Panayiotis M. Zavos, Cesare Aragona, Shady Abdelsattar Mohamed Ahmed Saleem, Maria Salomé Bezerra Espinola, Vanessa Alvarez Navarro

**Affiliations:** 1 Women Care and Fertility Center, Caracas 1080, Venezuela; 2 University of Kentucky, Lexington, KY 40504, USA; profzavos@zavos.org; 3 Università Degli Studi Unimarconi, 00193 Rome, Italy; aragonacesare@gmail.com; 4 Gynecology and In Vitro Fertilization, Arab Society of Fetal Medicine and Surgery, Cairo 11771, Egypt; drshady74@hotmail.com; 5 Marconi University, 00193 Rome, Italy; espinolasalome@gmail.com; 6 Santa Maria University, Caracas 1080, Venezuela; vanessaalv@outlook.es

**Keywords:** autologous exosomes, in vitro maturation (IVM), metaphase I oocytes, diminished ovarian reserve, assisted reproductive technologies (ART), oocyte maturation, extracellular vesicles, intracytoplasmic sperm injection (ICSI)

## Abstract

Oocyte development begins during embryonic life and remains arrested in prophase I until meiotic resumption during ovulation. A substantial proportion of oocytes retrieved during assisted reproductive procedures remain immature at the metaphase I (MI) stage and fail to achieve the maturation required for fertilization. In women with diminished ovarian reserve, alterations in the ovarian microenvironment may impair the biochemical signaling necessary for normal oocyte development. Autologous exosomes have emerged as a potential strategy to enhance meiotic progression through the transfer of bioactive molecules. This study aimed to evaluate the in vitro efficacy of autologous exosomes in improving the maturation potential of immature oocytes in women with diminished ovarian reserve. A prospective, randomized, controlled pilot study was conducted in 32 women aged 38–46 years. A total of 111 oocytes were retrieved (52 metaphase II [MII] and 59 MI oocytes), and only MI oocytes were included in the experimental analysis. MI oocytes were cultured either in conventional medium or in medium supplemented with autologous exosomes. The exosome-supplemented group demonstrated significantly higher in vitro maturation rates compared with the conventional culture group (63.63% vs. 11.53%; *p* < 0.001). Fertilization rates among matured oocytes were 76.19% and 33.33% in Groups II and I, respectively. In addition, favorable embryo development outcomes were observed in the exosome-treated group. Autologous exosome supplementation may enhance in vitro maturation and fertilization outcomes in women with diminished ovarian reserve. After accounting for clustering of oocytes within patients using generalized estimating equations, exosome supplementation remained significantly associated with successful oocyte maturation (OR 15.46, 95% CI 5.16–46.39; *p* < 0.001).

## 1. Introduction

Oocyte developmental competence is a key determinant of success in assisted reproductive technologies (ART). During folliculogenesis, the oocyte remains arrested in prophase I and resumes meiosis only in response to tightly coordinated endocrine and paracrine signals. In clinical practice, however, a substantial proportion of retrieved oocytes remain immature at the germinal vesicle (GV) or metaphase I (MI) stage, and only oocytes that reach metaphase II (MII) can be fertilized successfully [1,2,3,4]. Therefore, improving the in vitro maturation (IVM) of immature oocytes remains an important challenge, particularly in women with diminished ovarian reserve.

**Figure 1:**
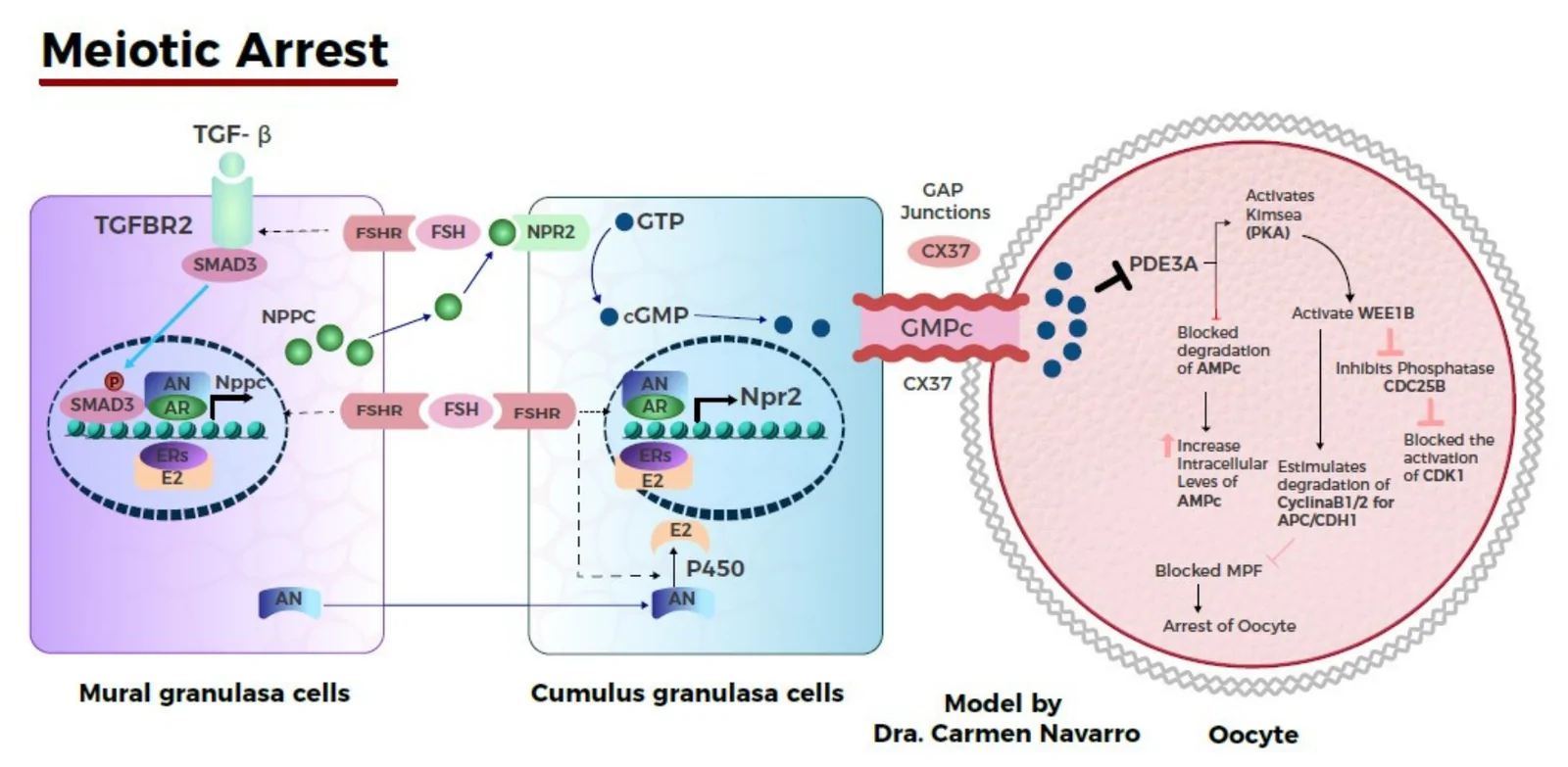
Schematic model representing the mechanisms of meiotic arrest of oocytes, adapted by Dr. Carmen Navarro.

The transition from meiotic arrest to meiotic resumption depends on a highly regulated biochemical microenvironment. Elevated intraoocyte cyclic adenosine monophosphate (cAMP), maintained through bidirectional communication between the oocyte and surrounding granulosa or cumulus cells, preserves meiotic arrest, whereas the preovulatory LH surge disrupts this balance and promotes progression to MII [3,4,5,6,7,8,9]. This process is modulated by signaling pathways involving NPPC/NPR2, EGFR-Ca^2+^ activation, gonadotropin responsiveness, and other regulatory mediators that coordinate both nuclear and cytoplasmic maturation (Figure 1) [10,11,12,13,14,15,16]. In women with ovarian aging or diminished ovarian reserve, these mechanisms may be impaired by alterations in the ovarian microenvironment, including impaired intercellular signaling and reduced availability of regulatory bioactive molecules [13,17,18,19].

**Figure 2:**
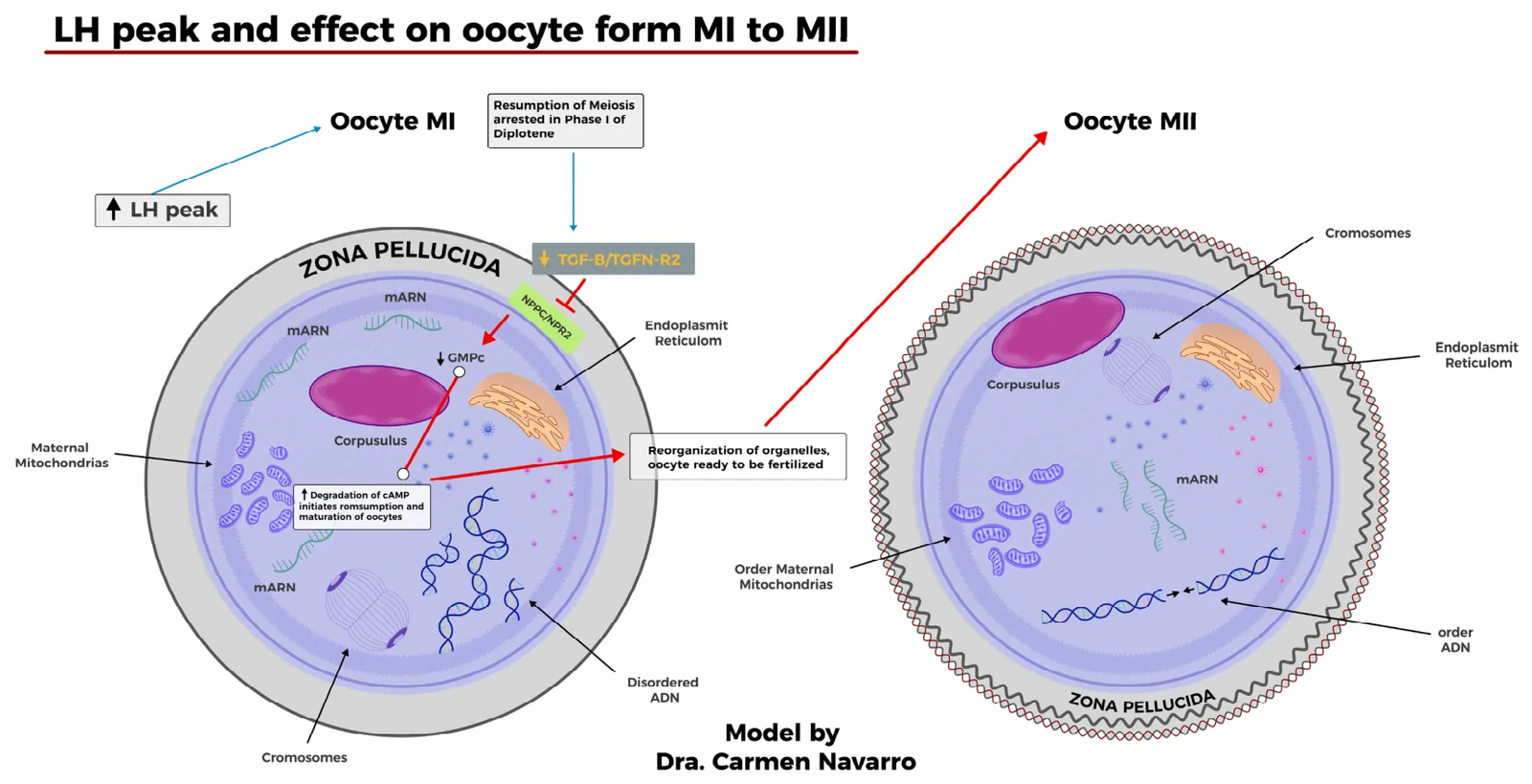
Schematic model of the intracellular reactions that lead to the maturation of the MI to MII oocyte secondary to the LH surge.

These biological limitations are especially relevant in patients with poor ovarian response, in whom the number of retrieved oocytes is already low and the proportion of immature oocytes may further compromise reproductive outcomes. Although IVM has evolved substantially over recent decades, conventional culture systems still provide suboptimal maturation rates in this subgroup of patients, suggesting the need for biologically active supplements capable of better reproducing the follicular microenvironment and supporting meiotic progression. Successful progression to the MII stage requires not only nuclear maturation but also coordinated cytoplasmic reorganization for developmental competence, including mitochondrial redistribution, endoplasmic reticulum reorganization, and cytoskeletal remodeling (Figure 2) [1,3,15,16].

Extracellular vesicles, particularly exosomes, have emerged as relevant mediators of intercellular communication in reproductive biology. Exosomes transport microRNAs, proteins, lipids, and other signaling molecules capable of modulating cellular metabolism, mitochondrial activity, cytoskeletal organization, and gene expression. In ovarian physiology, these vesicles may contribute to the biochemical dialogue required for oocyte maturation and developmental competence. In addition, available evidence regarding the differential microRNA content of extracellular vesicles derived from mature and immature oocytes supports the hypothesis that these mediators actively participate in the regulation of oocyte quality and reproductive potential (Table 1) [20,21].

Based on this biological rationale, autologous exosomes may represent a clinically attractive strategy in women with diminished ovarian reserve, as they provide patient-derived signaling cargo that could potentially compensate, at least in part, for the altered follicular microenvironment associated with meiotic arrest. In this context, the present prospective, randomized pilot study aimed to evaluate whether supplementation of conventional IVM medium with autologous exosomes could promote the maturation of human MI oocytes to the MII stage within 24–36 h of culture, as well as improve subsequent fertilization outcomes and embryo quality.

## 2. Objective of the Study

- To evaluate the ability of autologous exosomes to promote the meiotic progression of human oocytes from Metaphase I (MI) to Metaphase II (MII) during a 24–36 h in vitro culture period.
- To compare in vitro maturation (IVM), fertilization, and embryo quality rates between oocytes cultured in autologous exosome-supplemented media and those cultured in conventional media.
- To generate preliminary evidence regarding the potential of autologous exosomes as a complementary strategy to improve reproductive outcomes in women with diminished ovarian reserve and poor ovarian response.

## 3. Materials and Methods

This pilot prospective, randomized, controlled study was designed to evaluate the feasibility, safety, and preliminary biological efficacy of autologous exosome supplementation during in vitro maturation of oocytes obtained from infertile women with diminished ovarian reserve and poor ovarian response. The protocol was reviewed and approved by the Bioethics Committee of the Ministerio del Poder Popular para la Salud of Venezuela, ensuring compliance with current national and international regulations and the protection of participants’ rights in accordance with the principles of the Declaration of Helsinki for research involving human subjects. Patient recruitment was conducted prospectively between January 2023 and January 2025, during which participants were enrolled and followed in real time according to the study protocol.

### 3.1. Inclusion Criteria

- Women with primary or secondary infertility associated with diminished ovarian reserve, defined by ultrasound findings and laboratory parameters, including follicle-stimulating hormone (FSH) > 10 mIU/mL and estradiol < 60 pg/mL.
- Serum anti-Müllerian hormone (AMH) concentration < 1 ng/mL.
- Antral follicle count between 3 and 6 follicles per ovary.
- Anatomical preservation of both ovaries.
- Body mass index (BMI) between 24.5 and 30 kg/m^2^.
- Absence of active oncological diseases or connective tissue disorders.
- No history of clinically relevant hematological disorders.
- Adequate serum levels of vitamins essential for reproductive function, including vitamin D, vitamin B complex, and vitamin C.
- Last live birth within ≤10 years prior to study initiation.
- Normal thyroid function, including thyroid-stimulating hormone (TSH) and free thyroid hormone levels within normal ranges.
- Absence of antiphospholipid syndrome.
- Male partner with semen parameters within normal limits according to the World Health Organization (WHO) criteria, 2010 (5th edition) and 2021 (6th edition).
- Signed informed consent for participation in the study.

### 3.2. Exclusion Criteria

- Patients without a diagnosis of infertility.
- Women without documented diminished ovarian reserve.
- Serum anti-Müllerian hormone (AMH) concentration > 1 ng/mL.
- Antral follicle count > 6 follicles per ovary.
- Absence of one or both ovaries.
- Body mass index (BMI) > 30 kg/m^2^.
- Presence of active oncological diseases or connective tissue disorders.
- History of clinically relevant hematological disorders.
- Deficient or altered serum levels of vitamins essential for reproductive function, including vitamin D, vitamin B complex, and vitamin C.
- Last live birth > 10 years prior to study initiation.
- Altered thyroid function, including thyroid-stimulating hormone (TSH) or free thyroid hormone levels outside normal ranges.
- History of antiphospholipid syndrome.
- Male partner with abnormal semen parameters according to the World Health Organization (WHO) criteria, 2010 (5th edition) and 2021 (6th edition).
- Refusal to sign informed consent for participation in the study.

**Figure 3:**
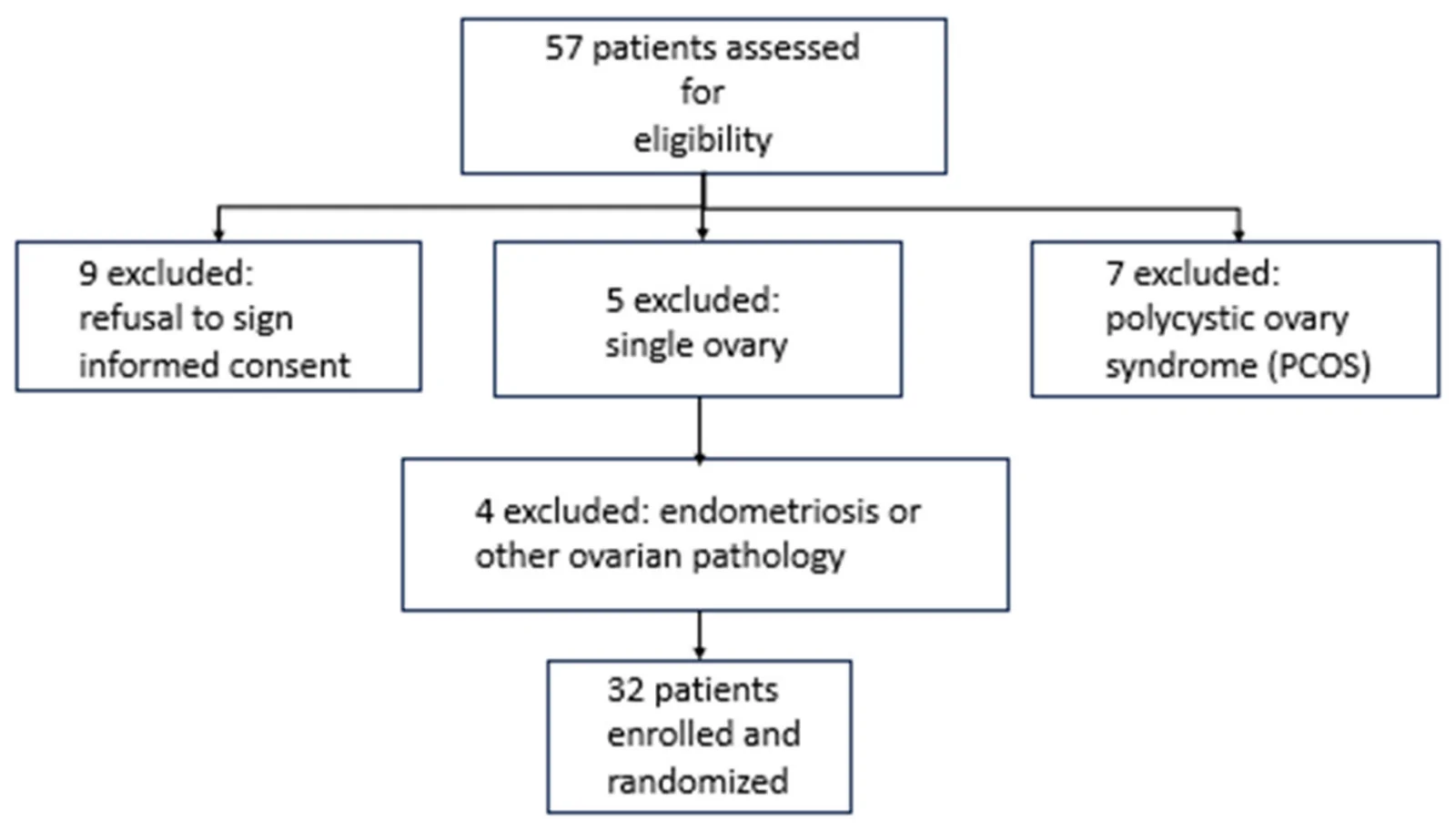
Distribution and selection of patients in the study.

A total of 57 infertile women aged between 38 and 46 years with diminished ovarian reserve were assessed for eligibility. Of these, 32 patients met the inclusion criteria and were enrolled and randomized in the study. All participants declined oocyte donation as a reproductive option (Figure 3).

All 32 selected patients underwent ovarian reserve assessment, including antral follicle count, serum anti-Müllerian hormone (AMH), follicle-stimulating hormone (FSH), estradiol levels, and reproductive history, including previous pregnancies and miscarriages (Table 2).

### 3.3. Ovarian Stimulation and Oocyte Retrieval

Thirty-two patients were included and randomized into two study groups, with 16 patients assigned to each group. All participants underwent a standardized ovarian stimulation protocol consisting of recombinant follicle-stimulating hormone (rFSH; Gonal-F®, Merck KGaA, Darmstadt, Germany) at a maximum dose of 300 IU/day for 10–12 days, combined with 150 IU/day of human menopausal gonadotropin (HMG; Merional®, IBSA Institut Biochimique S.A., Lugano, Switzerland) during the same period. Letrozole 5 mg/day was administered from day 1 to day 5 of ovulation induction. All patients received a gonadotropin-releasing hormone (GnRH) antagonist (Orgalutran®, Organon N.V., Oss, The Netherlands) once the follicles reached 14 mm in diameter, followed by administration of human chorionic gonadotropin (hCG; Ovidrel®, Merck KGaA, Darmstadt, Germany) to trigger final oocyte maturation.

Following oocyte retrieval, oocytes were classified according to their maturation stage as metaphase I (MI, immature) or metaphase II (MII, mature). A total of 111 oocytes were obtained, including 59 MI oocytes and 52 MII oocytes. All immature (MI) oocytes were included in the study and distributed according to the previously assigned study groups.

It is important to highlight the baseline distribution and characteristics of the retrieved oocytes in each randomized group prior to the allocation of MI oocytes to the corresponding in vitro maturation protocol. In Group I, in which immature oocytes were designated for culture in conventional IVM medium, a total of 43 oocytes were retrieved, including 26 MI oocytes and 17 MII oocytes. In Group II, in which immature oocytes were designated for culture in conventional IVM medium supplemented with 10 µg of autologous exosomes, a total of 68 oocytes were retrieved, including 33 MI oocytes and 35 MII oocytes.

**Figure 4:**
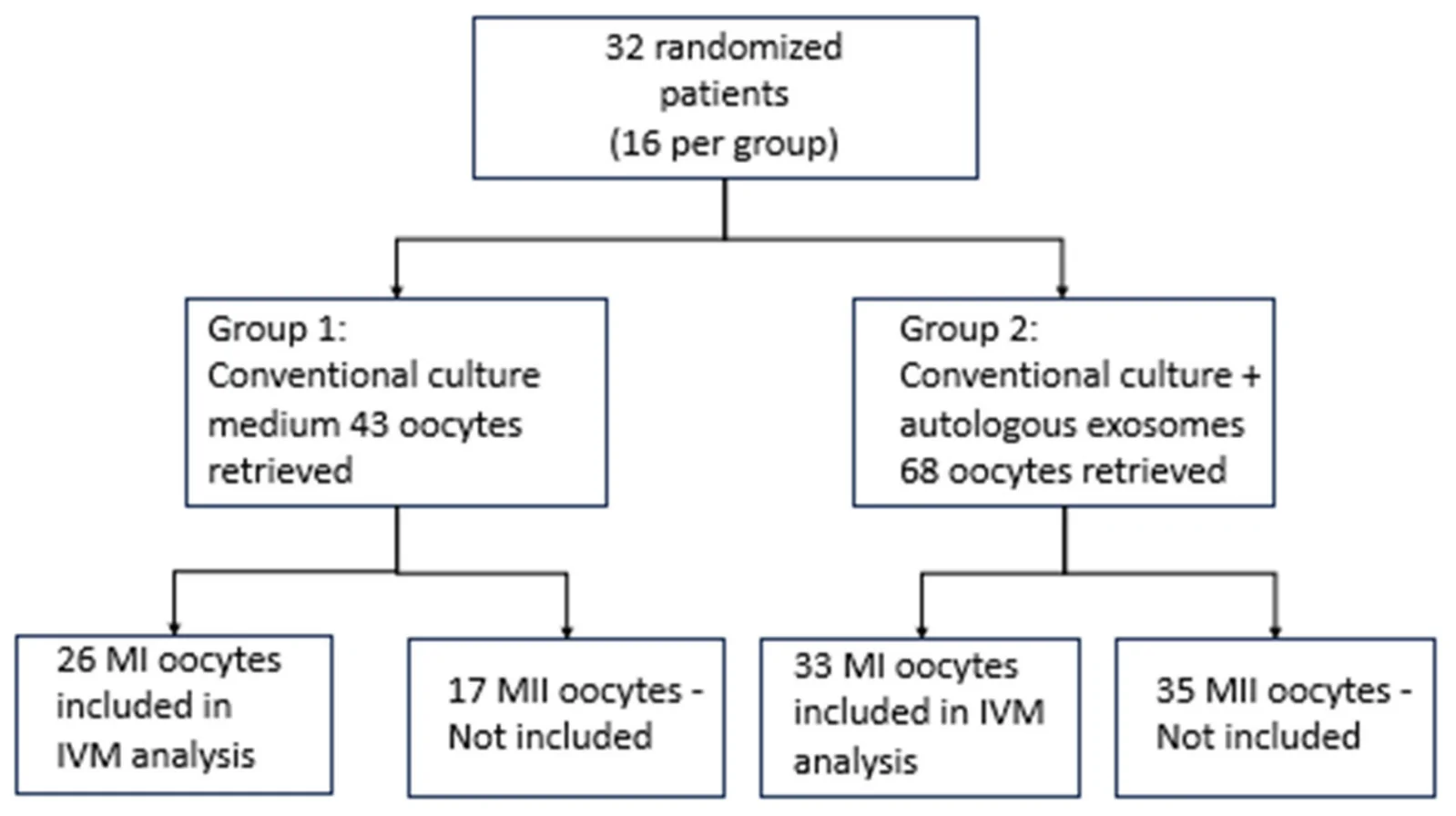
Classification and Distribution of Oocytes in the Study.

It should be emphasized that only MI oocytes were included in the experimental in vitro maturation analysis. Each oocyte was cultured individually and independently, maintaining complete traceability to the originating patient at all times and avoiding simultaneous culture or mixing of oocytes obtained from different participants. This methodological approach allowed preservation of the specific identification of each oocyte throughout all stages of the experimental procedure, from in vitro maturation to subsequent fertilization assessment and embryo development evaluation (Figure 4).

Following the previously established randomization process, patients were allocated into two study groups using a simple computer-generated randomization sequence created in Microsoft Excel (Microsoft Corporation, Redmond, WA, USA). Table 2 summarizes patient allocation, antral follicle count, total oocyte retrieval, and the distribution of metaphase I (MI) and metaphase II (MII) oocytes according to the study group. Baseline clinical, hormonal, and laboratory characteristics were evaluated prior to ovarian stimulation according to the predefined inclusion criteria (Table 3).

### 3.4. Study Groups

- Group I (Control): MI oocytes were cultured exclusively in conventional in vitro maturation (IVM) medium (Vitrolife®, Vitrolife AB, Gothenburg, Sweden).
- Group II (Experimental): MI oocytes were cultured in conventional in vitro maturation (IVM) medium (Vitrolife®, Vitrolife AB, Gothenburg, Sweden) supplemented with autologous exosomes obtained using the Exosmart® system (Medica S.p.A., Medolla, Modena, Italy) (Table 3).

The autologous exosomes used in this study were obtained from each patient’s peripheral blood. Following centrifugation of the blood sample using platelet-rich plasma (PRP) tubes with separator gel (RegenLab SA, Le Mont-sur-Lausanne, Switzerland) at 270× *g* for 10 min, approximately 40 mL of PRP was obtained. The centrifugation was performed using a horizontal swing-out centrifuge (Horizon, Drucker Diagnostics, Port Matilda, PA, USA) operating at 3500 rpm. Subsequently, the PRP was divided into two 20 mL syringes for processing using the Exosmart® autologous ultrafiltration system (Medica S.p.A., Medolla, Modena, Italy). After 5 min of ultrafiltration, approximately 4 mL of exosome suspension was obtained, from which a 10 µg aliquot was used to supplement the conventional culture medium of the oocytes included in the study.

The exosome dose used in this pilot study was selected as a biologically active working dose based on methodological reproducibility, viability in human metaphase I oocyte culture, and the available evidence regarding the potential effect of exosomes on oocyte maturation. MI oocytes included in each study group were evaluated after 24–36 h of culture, and the following findings were observed (Table 4).

Once the MII stage was achieved, all mature oocytes underwent intracytoplasmic sperm injection (ICSI). Fertilization was subsequently evaluated, and all embryos meeting acceptable morphological criteria, including the presence of 6–8 blastomeres, fragmentation < 35%, adequate morphology, and satisfactory expansion, were cryopreserved according to the informed consent previously provided and signed by each participant before enrollment in the study.

Due to the pilot and exploratory nature of the study, no embryo transfer was performed in any participant during this first phase of the investigation. This decision had been previously established in the informed consent and approved in the study protocol, considering the limited number of patients and embryos obtained, as well as the potential need to incorporate preimplantation genetic testing (PGT) prior to embryo selection and transfer in future phases of the study.

Although the immediate experimental unit corresponded to individual MI oocytes, patient traceability was maintained throughout the study, and results should be interpreted within the exploratory framework of this pilot design.

Although patients were randomized to the study groups, the primary outcome was evaluated at the oocyte level. Because multiple metaphase I (MI) oocytes could be obtained from the same patient, the statistical analysis accounted for the hierarchical structure of the data by considering the patient as the clustering unit.

### 3.5. Statistical Analysis

Statistical analysis was performed using IBM SPSS Statistics version 27.0 (IBM Corp., Armonk, NY, USA). Descriptive statistics were used to summarize the clinical, hormonal, and laboratory variables included in the study. Continuous variables were expressed as means ± standard deviation (SD) or medians and ranges, according to data distribution, whereas categorical variables were expressed as frequencies and percentages.

Baseline comparisons between study groups were performed using parametric or non-parametric statistical tests, as appropriate according to the characteristics and distribution of the variables analyzed. Because multiple metaphase I (MI) oocytes could originate from the same patient, individual oocytes were not considered statistically independent observations. Therefore, the primary inferential analysis evaluating oocyte maturation was performed using generalized estimating equations (GEE) with a binomial distribution, logit link function, and an exchangeable working correlation structure, specifying the patient as the clustering variable, as recommended for correlated binary outcomes arising from clustered observations [22,23]. Effect estimates were expressed as odds ratios (ORs) with corresponding 95% confidence intervals (95% CI). Secondary outcomes, including fertilization and Day 3 embryo cryopreservation, were analyzed descriptively because of the exploratory pilot nature of the study. Statistical significance was established at a two-sided *p*-value < 0.05. Given the pilot exploratory nature of the study, no formal sample size calculation was performed.

## 4. Results

A total of 32 patients were included in the final analysis. The mean age of the participants was 42.16 ± 2.63 years, with most patients between 40 and 45 years of age. Regarding reproductive history, 56.3% of participants had a previous pregnancy, while 43.8% were nulliparous. Baseline sociodemographic and obstetric characteristics are summarized in Table 5.

The primary outcome analysis demonstrated a statistically significant difference in oocyte maturation rates between the control and exosome-supplemented groups (*p* < 0.05), with higher maturation rates observed in the exosome-treated group (Table 5).

Among the 24 oocytes that achieved in vitro maturation to the MII stage (3 from Group I and 21 from Group II), 17 were successfully fertilized, resulting in an overall fertilization rate of 70.83%. Fertilization rates were higher in the exosome-supplemented group compared with the conventional culture group (76.19% vs. 33.33%, respectively), with statistically significant differences observed between groups (*p* < 0.001; Table 6).

The MI oocytes that reached metaphase II (MII) after 24–36 h of in vitro maturation underwent intracytoplasmic sperm injection (ICSI). Embryos were morphologically evaluated on Day 3 according to predefined criteria, including 6–8 blastomeres, cellular uniformity, and fragmentation < 30%. A total of 12 embryos from the exosome-supplemented group met the established criteria for cryopreservation.

The exosome-supplemented group demonstrated higher rates of in vitro maturation and fertilization compared with the conventional culture group. The maturation rate was significantly higher in the exosome group (63.63% vs. 11.53%; *p* < 0.001), while fertilization rates among oocytes that achieved MII maturation were 76.19% and 33.33% for Groups II and I, respectively. In addition, 75% of embryos obtained in the exosome-supplemented group met predefined morphological quality criteria for cryopreservation.

Because multiple immature oocytes originated from the same patient, a generalized estimating equation (GEE) model was fitted to account for within-patient correlation. After adjustment for clustering, autologous exosome supplementation remained independently associated with successful oocyte maturation (OR 15.46, 95% CI 5.16–46.39; Wald χ^2^ = 23.86; *p* < 0.001) (Table 7).

## 5. Discussion and Future Perspectives

Our findings suggest that supplementation of the IVM culture medium with autologous exosomes may enhance the meiotic progression of immature MI oocytes in women with diminished ovarian reserve. Rather than merely increasing the number of oocytes reaching the MII stage, this effect appears to be accompanied by preservation of post-maturation developmental competence, as reflected by the subsequent fertilization performance and embryo development observed in this pilot series. These findings are clinically relevant because patients with diminished ovarian reserve often have a limited number of retrievable oocytes, and any strategy capable of improving the usability of immature oocytes may have meaningful reproductive value. Recent reviews on human oocyte IVM emphasize that immature oocytes recovered during ART remain an important underused biological resource, particularly in patients with poor ovarian response, but that current IVM systems still require optimization to improve clinical applicability [24,25].

A plausible biological explanation for this effect is the role of exosomes as mediators of intercellular communication within the ovarian microenvironment. Extracellular vesicles transport microRNAs, proteins, lipids, and signaling molecules that can regulate mitochondrial activity, oxidative balance, cytoskeletal dynamics, and gene expression, all of which are essential for both nuclear and cytoplasmic maturation of the oocyte. In the context of ovarian aging or diminished ovarian reserve, where follicular signaling may be impaired, autologous exosomes may partially restore a deficient biochemical microenvironment and thereby facilitate meiotic resumption. Recent reviews specifically focused on oocytes highlight extracellular vesicles as emerging regulators of oocyte quality and maturation, supporting their relevance as a biologically grounded therapeutic strategy [19].

Our results are also consistent with previous experimental evidence suggesting that exosome supplementation may improve oocyte maturation under in vitro conditions. Han et al. demonstrated that follicular fluid-derived exosomes enhanced porcine oocyte maturation and mitochondrial membrane potential, supporting the concept that vesicle-mediated signaling can improve oocyte competence during IVM. Similarly, Ren et al. showed that granulosa-cell-derived exosomes enhanced porcine oocyte development and antioxidant capacity, further reinforcing the view that exosomes can actively modulate the oocyte microenvironment and developmental potential. Although these data originate from animal models, they provide mechanistic support for the present findings and strengthen the rationale for testing exosome-based supplementation in human IVM systems [21,26].

The beneficial effect of autologous exosomes remained statistically significant after accounting for the correlation among multiple oocytes obtained from the same patient using generalized estimating equations. This analytical approach is recommended when clustered observations violate the assumption of independence required by conventional regression models and provides more reliable population-averaged effect estimates [22,23].

Nevertheless, these findings should be interpreted with caution. This was a pilot study with a limited sample size, and the study was not designed to establish definitive clinical effectiveness. In addition, although improved maturation and early developmental outcomes are encouraging, they do not by themselves demonstrate reproductive competence in terms of implantation, clinical pregnancy, or live birth. Current reviews of human IVM continue to emphasize that the field is limited by variability in laboratory protocols, patient selection, and culture conditions, and that future progress will depend on better standardization and mechanistic characterization. In this regard, future studies should incorporate larger cohorts, dose–response assessment, detailed characterization of exosome cargo, and reproductive endpoints beyond early embryo development. In addition, advanced physicochemical characterization of exosome preparations, including nanoparticle tracking analysis (NTA), transmission electron microscopy (TEM), and exosomal marker profiling, was not performed in this pilot phase and should be incorporated in future translational studies [24,25].

Taken together, our study supports the hypothesis that autologous exosomes may serve as a biologically relevant supplement for IVM in patients with diminished ovarian reserve. The present results should be considered preliminary but promising, as they suggest that patient-derived extracellular vesicles may improve the maturation efficiency of MI oocytes while preserving subsequent developmental potential. Further translational and clinical validation will be necessary before this strategy can be incorporated into routine reproductive practice.

### Limitations

Although generalized estimating equations accounted for the correlation among oocytes from the same patient, the relatively small number of women included limits the precision of the estimated treatment effect and these findings should be confirmed in larger randomized controlled trials.

## 6. Conclusions

This pilot study suggests that supplementation of conventional IVM medium with autologous exosomes may enhance the in vitro maturation of metaphase I (MI) oocytes in women with diminished ovarian reserve. Higher maturation and fertilization rates were observed in the exosome-supplemented group, together with favorable early embryo development outcomes.

Although these findings are preliminary and should be interpreted within the exploratory nature of the study, they support the potential role of autologous exosomes as a biologically relevant adjunctive strategy for human oocyte IVM. Further studies with larger sample sizes, standardized exosome characterization, and reproductive outcome assessment will be necessary to validate the clinical applicability of this approach.

## Figures and Tables

**Table 1:** Content of different types of microRNAs in extracellular vesicles from mature and immature oocytes in humans and animal models, determining higher success rate in reproduction.

Species	Upregulated Cargo	Downregulated Cargo	Oocyte Quality Criteria	Ref.
Human	miR-214 and miR-454	miR-92 a, miR-130 b, and miR-888	Fertilization status and embryo quality status	[19]
Human	miR-1246, miR-548 ae-5 p, miR-505-3 p, miR-548 t-3 p, miR-548 au-5 p, miR-320 e, and miR-1303	miR-513 c-5 p and miR-548 au-3 p	Antral follicle count and AMH level	[19]
Porcine	miR-125 b, miR-193 a-5 p, and miR-320	miR-9, miR-206, and miR-6516	Oocyte stain	[21]
Bovine	25 lipids including DAG 36:0 + NH4, TAG 56:0 + NH4, NAPE 40:1 + NH4, etc.	–	The capability of the oocyte to develop to the blastocyst stage	[19]
Human	mRNA of ETC complex I subunit 1, ETC complex III cytochrome b	–	Fertilization status and embryo quality status	[19]

**Table 2:** Baseline clinical and hormonal characteristics of patients included in the study according to randomized study group.

Patient ID	Study Group	Antral Follicle Count (Both Ovaries)	AMH: Anti-Müllerian Hormone (ng/mL)	FSH: Follicle-Stimulating Hormone (mIU/mL)	Estradiol (pg/mL)	Previous Pregnancy	Number of Previous Pregnancies	Previous Miscarriages
EX001	Experimental	6	1	10.6	54	Yes	2	0
EX002	Control	3	0.5	14.1	52	Yes	3	1
EX003	Experimental	5	0.8	11.6	54	No	0	0
EX004	Experimental	2	0.3	19	39	Yes	2	0
EX005	Experimental	2	0.2	21	30	No	0	0
EX006	Control	3	0.6	13.8	49	No	0	0
EX007	Control	6	1	11.2	54	Yes	1	0
EX008	Experimental	5	0.9	14.3	53	Yes	1	0
EX009	Control	6	0.9	12.5	54	Yes	2	0
EX010	Experimental	5	1	10.8	53	No	0	0
EX011	Experimental	3	0.8	13.5	40	No	0	0
EX012	Control	3	0.5	22.1	32	Yes	2	0
EX013	Experimental	5	0.7	16.2	53	Yes	1	0
EX014	Control	6	0.9	11.4	54	Yes	3	0
EX015	Control	4	0.2	27.2	32	No	0	0
EX016	Experimental	6	0.8	14.2	54	No	0	0
EX017	Control	5	1	11.5	52	Yes	2	0
EX018	Experimental	6	0.8	10.8	53	Yes	1	0
EX019	Control	4	0.9	18.1	39	Yes	2	1
EX020	Control	3	0.3	15.3	31	No	0	0
EX021	Experimental	6	1	10.3	54	Yes	1	0
EX022	Control	5	0.8	12.1	53	Yes	2	0
EX023	Experimental	6	1	11.67	48	Yes	3	1
EX024	Experimental	4	1	13	41	No	0	0
EX025	Control	3	0.3	18	33	No	0	0
EX026	Experimental	6	1	12.1	59	Yes	2	0
EX027	Control	4	0.3	15	60	No	0	0
EX028	Experimental	3	0.9	12	59	Yes	1	0
EX029	Control	2	0.2	22	35	No	0	0
EX030	Experimental	5	0.6	14	60	Yes	2	0
EX031	Control	3	0.4	17	38	No	0	0
EX032	Control	2	0.5	30	25	No	0	0

**Table 3:** Patient allocation, antral follicle count, total oocyte retrieval, and distribution of metaphase I (MI) and metaphase II (MII) oocytes according to study group randomization.

Patient	Age	Antral Follicle Count in Both Ovaries	Total of Oocites Collected	Oocites MI	Oocites MII	Random Group
EX001	39	6	5	2	3	2
EX002	43	3	3	2	1	1
EX003	38	5	5	1	4	2
EX004	45	2	2	2	0	2
EX005	46	2	2	1	1	2
EX006	44	3	2	1	1	1
EX007	39	6	5	2	3	1
EX008	40	5	5	3	2	2
EX009	40	6	4	2	2	1
EX010	38	5	5	3	2	2
EX011	44	3	2	1	1	2
EX012	45	3	2	2	0	1
EX013	43	5	3	2	1	2
EX014	41	6	5	1	4	1
EX015	46	4	1	1	0	1
EX016	42	6	6	2	4	2
EX017	40	5	4	2	2	1
EX018	42	6	5	3	2	2
EX019	44	4	3	2	1	1
EX020	45	3	1	1	0	1
EX021	39	6	6	3	3	2
EX022	42	5	5	3	2	1
EX023	41	6	6	3	3	2
EX024	38	4	3	1	2	2
EX025	43	3	1	0	1	1
EX026	39	6	6	3	3	2
EX027	45	4	3	3	0	1
EX028	41	3	3	1	2	2
EX029	46	2	1	1	0	1
EX030	42	5	4	2	2	2
EX031	43	3	2	2	0	1
EX032	46	2	1	1	0	1

**Table 4:** In vitro maturation of metaphase I (MI) oocytes after 24–36 h according to study group.

Group	Total MI Oocytes	Matured MI Oocytes (MII), *n*	Maturation Rate (%)	*p*-Value
Group I (conventional culture medium)	26	3	11.53	<0.001
Group II (conventional culture medium + autologous exosomes)	33	21	63.63	

**Table 5:** Baseline sociodemographic, reproductive, and ovarian reserve characteristics of the participants.

Variable	Category/Parameter	Value
Age (years)	Mean ± SD	42.16 ± 2.63
Age group	<40 years	7 (21.9%)
	40–45 years	21 (65.6%)
	>45 years	4 (12.5%)
Parity status	Nulliparous	14 (43.8%)
	Non-nulliparous	18 (56.3%)
Number of previous pregnancies	1 pregnancy	6 (18.8%)
	2 pregnancies	9 (28.1%)
	3 pregnancies	3 (9.4%)
Previous miscarriages	Yes	3 (9.4%)
	No	29 (90.6%)
Antral follicle count	Mean ± SD	4.28 ± 1.44
AMH (ng/mL)	Mean ± SD	0.69 ± 0.29
FSH (mIU/mL)	Mean ± SD	15.19 ± 4.85
Estradiol (pg/mL)	Mean ± SD	46.78 ± 10.34

*AMH: anti-Müllerian hormone; FSH: follicle-stimulating hormone-N = 32 patients.*

**Table 6:** Fertilization outcomes and embryo development after in vitro maturation according to study group.

Group	Fertilized MII Oocytes (ICSI), *n*	Fertilization Rate (%)	Day 3 Embryos, *n*	Embryos Suitable for Cryopreservation, *n*	Cryopreservation Rate (%)	*p*-Value
Group I	1	33.33	1	1	100	<0.001
Group II	16	76.19	16	12	75	

**Table 7:** Generalized estimating equation analysis of oocyte maturation.

Variable	OR	95% CI	Wald χ^2^	*p*-Value
Autologous exosome supplementation	15.46	5.16–46.39	23.86	<0.001

## Data Availability

Due to the exploratory and pilot nature of the protocol, embryo transfer was intentionally not performed in any participant during this first phase of the investigation. This decision was previously established in the informed consent and approved by the ethics committee, considering the limited number of patients and embryos obtained, as well as the potential need to incorporate preimplantation genetic testing (PGT) prior to future embryo selection and transfer. Consequently, all embryos meeting predefined morphological quality criteria were cryopreserved for potential future evaluation within subsequent study phases.
